# The changing epidemiology of esophageal cancer in sub-Saharan Africa – the case of Ghana

**Published:** 2012-09-07

**Authors:** Mark Tettey, Frank Edwin, Ernest Aniteye, Lawrence Sereboe, Martin Tamatey, Ernest Ofosu-Appiah, Innocent Adzamli

**Affiliations:** 1 National Cardiothoracic Center, Box KB 846 Korle Bu Teaching Hospital, Accra, Ghana

**Keywords:** Esophageal cancer, squamous cell carcinoma, adenocarcinoma, dysphagia

## Abstract

**Introduction:**

Esophageal cancer portends a grim prognosis. Most patients present with incurable disease. Scanty epidemiologic data on the disease has contributed to its low priority on the national. We sought to evaluate the current national trend in the presentation and outcome of esophageal cancer using our institutional experience from 1992 – 2010.

**Methods:**

This is a retrospective study based on 152 patients who were seen in our institution during the study period. The perioperative data of these patients were retrieved and the relevant details recorded. Histopathological reports were available for 75 patients managed over the period. The study setting was The National Cardiothoracic Centre, which serves as the only tertiary referral centre in the country for cardiothoracic problems.

**Results:**

There were 122 males and 30 females with a mean age of 57.8±11.7 years. The yearly trend from 1992 to 2010 showed a steady increase in the incidence of esophageal cancer. High alcohol consumption and smoking dominated the history of 82.2% of the patients. Squamous cell carcinoma accounted for 78.7% and adenocarcinoma 21.3%. Distribution of esophageal carcinoma by anatomical location was 84.9% for distal third, 11.8% for middle third and 3.3% for upper third. All patients presented with incurable disease.

**Conclusion:**

The study shows an increasing incidence of esophageal carcinoma in this country. Alcohol abuse and smoking are major risk factors; squamous cell carcinoma is the dominant histological type in this study.

## Introduction

The esophagus, a muscular tube measuring 20-25 cm long and 2-3cm wide serves as a conduit for food and drink. Structurally the esophageal wall is composed of five histological layers: superficial mucosa layer, lamina propria, submucosa, muscularis propria and adventitia. The esophagus descends anterior to the vertebral column through the superior and posterior mediastinum. After traversing the diaphragm at the diaphragmatic hiatus the esophagus extends through the gastroesophageal junction to end at the gastric cardia [1]. Significantly, the esophagus is inaccessible to clinical examination. Clinical diagnosis of an esophageal lesion is thus based on symptoms and imaging studies. Carcinoma of the esophagus starts innocuously in the esophageal mucosa as a painless lesion and progresses to an advanced lesion before symptoms become apparent. The most common presentation of esophageal cancer leading to its diagnosis is progressive dysphagia. The esophagus is capable of accommodating the initial obstruction because it lacks a serosal layer which allows the smooth muscle to stretch. As a result the patient may not manifest dysphagia until the lumen is more than 50 - 60% obstructed by the tumor.

Esophageal cancer is a devastating disease that continues to have less than 10% five year survival despite advances in multimodality therapy [2]. It is the 8th most common malignancy worldwide and the sixth most common cause of cancer-related deaths [3]. This is the only cancer that is recording an increasing incidence all over the world in spite of the treatment modalities currently available for the management of this disease. The incidence of esophageal cancer represents one of the widest variations with a 60 - fold difference between high and low-incidence regions [4]. It is endemic in many parts of the world particularly in developing countries. High prevalence areas include Asia, Southern and Eastern Africa, and Northern France [4].

Racial disparities in esophageal cancer incidence, mortality and histology exist [5]. Esophageal cancer was reported as the 4th leading cause of death in African Americans [2]. The incidence of esophageal squamous cell carcinoma is more than fivefold higher among blacks than among whites in the USA [2]. The prevalence of squamous cell carcinoma in Africa is higher than adenocarcinoma and these epidemiologic observations are important because of the potential contributions to the understanding of the etiology and pathogenesis of esophageal cancer [2]. The purpose of this study is to evaluate the current national trend in the presentation and outcome of esophageal cancer using our institutional experience spanning two decades. The risk factors and the management options adopted in the treatment of the disease are also reviewed.

## Methods

We retrospectively reviewed all patients who were treated at Ghana's National Cardiothoracic Centre from 1992 to 2010. Data collected from patients’ case notes included age, sex, duration of symptoms, history of alcohol ingestion and cigarette smoking, staging of extent of disease surgical procedures performed and intraoperative findings for those who underwent surgery. Information regarding the histopathological diagnosis for some of these patients was also recorded.

### Statistical Analysis

Continuous data are represented as mean with standard deviation. Categorical data are represented in frequency and proportions. Chi square test was performed to compare the type of carcinoma and the regional distribution in the esophagus.

## Results

**Figure 1:**
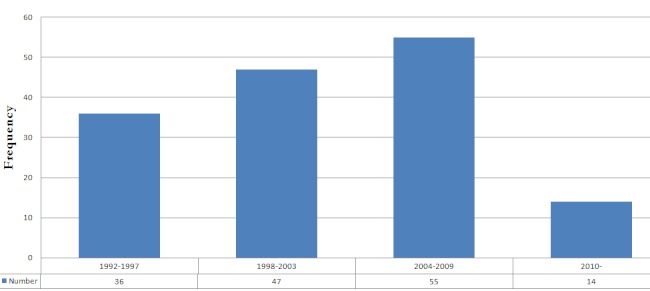
Incidence of esophageal carcinoma

Over the 18 year period, 152 patients with carcinoma of the esophagus were seen. Of these, 122 (80.3%) were males and 30 (19.7%) were females. The age distribution is shown in Table 1. The patients’ age range was 27 to 87 years with mean age of 57.8±11.7 years. The data show an increasing incidence of esophageal carcinoma since 1992. This is shown in Figure 1.

**Table 1:** Age distribution of the 152 patients

Age(years)	Number of patients	percentage
20 – 29	2	1.3
30 – 39	9	5.9
40 – 49	28	18.4
50 – 59	43	28.3
60 – 69	42	27.6
70 – 79	22	14.5
>80	6	4.0
**Total**	**152**	**100**

The distribution of esophageal cancer by anatomical location was 5 (3.3%) in the upper third, 18(11.8%) in the middle third and 129 (84.9%) in the distal third. Analyzing 75 patients from the 152 patients in whom histopathological information was available, 78.7% had squamous cell carcinoma and 21.3% had adenocarcinoma. Table 2 shows the histopathological distribution of the lesions. The type of carcinoma and the regional distribution was not significant in this study, p= 0.096.

**Table 2:** Histopathological distribution of cancer of the esophagus

	Adenocarcinoma	Squamous cell carcinoma	Total
Upper Third		2	2
Middle Third		12	12
Distal Third	16	45	61
**Total**	**16**	**59**	**75**
%	21.3%	78.7%	100%

One hundred and twenty five patients (82%) had history of alcohol ingestion and smoking. Of the 16 cases of adenocarcinoma, 11 (68.8%) had no history of alcohol or smoking; of the 59 cases of squamous cell carcinoma, 6 (10.1%) had no history of alcohol or smoking. These six cases of squamous cell carcinoma occurred exclusively in the middle third of the esophagus. The distribution of squamous cell carcinoma was 45 (76.3%) distal third, 12 (20.3%) middle third and 2 (3.4%) upper third. The most common presentation of esophageal cancer leading to diagnosis was a short history of progressive dysphagia. Sixty-three percent of the patients studied had 1 - 4 months’ history of progressive dysphagia while the duration was 5 - 10 months in the rest. Liver metastasis was present in 40 (36.2%) and lung metastasis in 6 (5.4%) of the patients studied. The type of surgical procedure performed is shown in Table 3. All patients presented with incurable disease. Ninety one (59.9%) of these patients had inoperable tumours; 61 (40.1%) presented with resectable but incurable tumors. Most patients were lost to follow up.

**Table 3:** Type of Surgical Procedure performed

Procedure	Number	Percentage
Feeding Gastrostomy	32	34.2
Esophagoscopy only	3	2.0
Esophagectomy with Intrathoracic Esophagogastrostomy	47	31.0
Esophagectomy with Colon interposition	14	9.2
Colon bypass without Esophagectomy	18	11.8
Stenting	38	11.8
**Total**	**152**	**100**

## Discussion

The mean age at diagnosis of esophageal cancer in the current study was 58.4 years with an age range of 27 - 87 years. The bulk of the disease presented in the 50 - 59 year group (Table 1). This is similar to the modal age in a retrospective study carried out in Kenya [6]. The mean age of 3,319 esophageal cancer patients in California was 63.8 years [7]. In Northern Iran where squamous cell carcinoma was not common, the mean age was 61.8±12.0 years [8]. In a systematic review of esophageal cancer in Sub-Saharan Africa, the disease was prominent among the age group of 45 - 65 years in both sexes [9]. In both developed and developing countries the modal age at presentation of esophageal carcinoma appears to be similar. Both squamous cell carcinoma and adenocarcinoma of the esophagus are infrequent before 40 years of age, beyond which the incidence of each type rises with each decade of life. [2] Most of these patients have identifiable risk factors that could be linked with the genesis of the esophageal disease. However, in a recent study in Western Kenya with very high rates of esophageal cancer, an unusual percentage of cases were found in subjects 30 years of age and younger [10]. Majority of these patients had a family history of cancer.

The sex ratio (male to female) all over the world shows significant diversity ranging from 0.85 in Northern Iran [8] to as high as 20.5 in Hispanics [11]. The male to female ratio in our study was 4:1. Other studies have recorded varying figures. [7, 9, 10, 12, 13] In a study by Nordenstedt and Seraq, the male to female ratio of esophageal cancer varied according to histology, age and race [11]. In their report, the results showed that for esophageal adenocarcinoma, all races had similar sex- and age- specific incidence patterns with a peak in the male to female ratio in the age group 50 -59 years [11]. The highest male to female ratio was seen in Hispanics (20.5) and the lowest in Blacks (7.0) compared with (10.8) in whites. By contrast the male to female ratios were low and fairly stable throughout the different age groups in esophageal squamous cell carcinoma [11].

The incidence of esophageal cancer has shown a steady increase over the years in this study. The conclusion from the review of esophageal cancer in Sub - Saharan Africa clearly shows that esophageal cancer incidence is on the ascendancy in the sub-region [9]. This is in keeping with our findings and the call for esophageal carcinoma to be included among the diseases of public health importance for effective prevention, early diagnosis and effective treatment is therefore valid.

The most common histopathological type in our study was squamous cell carcinoma (78.7%). This percentage is lower than that in studies from Kenya and South India where over 90% of patients diagnosed with esophageal cancer had squamous cell carcinoma [6, 14]. In a study of esophageal cancer epidemiology in blacks and whites, squamous cell carcinoma was more commonly diagnosed in blacks and white females whereas adenocarcinoma was more common among white males [5]. In a study among patients =30 years with esophageal cancer in Kenya, squamous cell carcinoma accounted for 98% of the cases [10]. Less than 20% of these patients took alcohol or smoked cigarette. The peculiarity of squamous cell carcinoma among blacks both in and out of Africa cannot be related to alcohol, smoking and social class alone. As stated by Brown et al in their study, the higher incidence rates observed among blacks for exposure to the same risk factors as whites may reflect a susceptibility state conditioned by genetic traits [15]. There should be some genetic bases to explain this especially when white females have similar predisposition as in blacks.

In Western cultures, retrospective evidence has implicated cigarette smoking and chronic alcohol exposure as the most common etiological factors for squamous cell carcinoma [16]. In this study 82% of the patients took alcohol and smoked cigarette in significant quantities. Tobacco and/or alcohol are known to account for approximately 90% of all esophageal squamous cell carcinoma [17]. Tobacco is linked to esophageal adenocarcinoma but there is no scientific evidence linking alcohol to esophageal adenocarcinoma [17]. Already known is the risk of alcohol and smoking in the causation of esophageal squamous cell carcinoma [3, 4, 18, 19]. Recent studies have shown that alcohol consumption and tobacco use do not affect the risk of esophageal carcinoma in the same way. For alcohol consumption, it is the mean intake (>200g/week) rather than the duration, whereas for tobacco smoking, it is the duration (>15 years) rather than the mean intake that is more closely associated with the risk of esophageal carcinoma [20]. However, the joint effect of alcohol and smoking when consumed together are potentiated and the final relative risk is multiplied [19]. Eighty nine percent of our patients were from low socioeconomic class. Socioeconomic class appears to be an independent risk factor in the development of esophageal carcinoma [2]. The combination of all four risk factors - low social class, moderate/heavy alcohol intake, and infrequent consumption of raw fruits and vegetables - accounted for almost all of the squamous cell carcinoma of the esophagus in one study [15].

The distribution of esophageal cancer by anatomical location was 129 (84.9%) in the distal third, 18 (11.8%) in the middle third and 5 (3.3%) in the upper third. Analyzing 75 of the patients from the 152 patients whose histopathological information were available, 78.7% had squamous cell carcinoma and 21.3% had adenocarcinoma. The distribution of squamous cell carcinoma was 45 (61%) in the distal third, 12 (33%) in the middle third and 2 (6%) in the upper third. Esophageal adenocarcinoma is exclusively a distal third tumor as was the result in this study. The type of carcinoma and the distribution was not significant in this study, p = 0.097. This is because in our study, squamous cell carcinoma is more common in the distal third of the esophagus. The diversity of the distribution of esophageal squamous cell carcinoma is demonstrated as some studies have shown that squamous cell carcinoma primarily occurs more in the middle third [2, 20]. Other studies are in keeping with our findings where lower esophageal cancers outnumbered the mid esophageal squamous cell carcinoma [8, 14]. Interestingly, patients who developed squamous cell carcinoma who neither drank alcohol nor smoked cigarette had their tumor exclusively located in the middle third of the esophagus in this study.

Similar to other studies [16, 20–22] we found the most common presentation leading to diagnosis of esophageal cancer was a short history of progressive dysphagia. Sixty three percent of our patients presented with 1 - 4 months history of progressive dysphagia. The absence of serosa and the distensibility of the esophagus delay the symptoms of esophageal cancer until the tumor is advanced. This could contribute to the variable duration of symptoms [6].

The distant nodal disease defined as M1a by TNM staging system could be assessed in 111 patients. Out of these patients 74 (66.7%) had evidence of distant node involvement located around the celiac and/or para-aortic areas. It has been shown that having even one lymph node positive for disease decreases survival rate by 25 - 40% and having more than 4 positive nodes is associated with survival rates of <5% [23]. The distant organ metastasis using chest X-ray, computerized tomography scan, ultrasound scan and intra-operative assessment showed distant metastasis in the liver in 40 (36%) patients and in the lung in 6 (5.4%) patients. PET scan is more accurate in detecting distant metastasis [24, 25] though this was not used in this study.

Esophageal cancer generally portends a grim prognosis attributable to late presentation in most patients and all patients in this study presented with incurable disease. Sixty one percent of these patients had inoperable tumors and 39% although resectable were incurable. Patients in this study showed no survival benefit from neoadjuvant therapy. Some underwent initial feeding gastrostomy to prepare for neoadjuvant therapy but most of them deteriorated and could not undergo surgery. Esophageal stenting was done for patients with metastatic disease. In a report by Patel et al, there was no advantage of neoadjuvant therapy over surgery alone [23]. We employed adjuvant chemoradiation after palliative esophagectomy in some selected patients. However, postoperative adjuvant therapy has not been shown to be beneficial [26]. We decided to bypass some selected cases (without metastases, comorbidities, or severe nutritional deficiency) of inoperable tumors with colon and then refer them for adjuvant chemotherapy. Most of these patients did well until their demise from locally advanced or metastatic disease.

## Conclusion

The national trend of esophageal carcinoma shows an increasing incidence over the past 2 decades. Alcohol consumption and smoking are major risk factors. Squamous cell carcinoma is the dominant histological type. Late presentation characterized most patients; conventional management did not impact on the survival. Early diagnosis and treatment is advocated but remains an uphill task.
